# Dual Role of ROS as Signal and Stress Agents: Iron Tips the Balance in favor of Toxic Effects

**DOI:** 10.1155/2016/8629024

**Published:** 2016-02-24

**Authors:** Elena Gammella, Stefania Recalcati, Gaetano Cairo

**Affiliations:** Department of Biomedical Sciences for Health, University of Milan, 20133 Milan, Italy

## Abstract

Iron is essential for life, while also being potentially harmful. Therefore, its level is strictly monitored and complex pathways have evolved to keep iron safely bound to transport or storage proteins, thereby maintaining homeostasis at the cellular and systemic levels. These sequestration mechanisms ensure that mildly reactive oxygen species like anion superoxide and hydrogen peroxide, which are continuously generated in cells living under aerobic conditions, keep their physiologic role in cell signaling while escaping iron-catalyzed transformation in the highly toxic hydroxyl radical. In this review, we describe the multifaceted systems regulating cellular and body iron homeostasis and discuss how altered iron balance may lead to oxidative damage in some pathophysiological settings.

## 1. Introduction

In eukaryotes, oxygen utilization by the mitochondria, NADPH oxidase enzymes, cytochromes, and so forth leads to the generation of reactive oxygen species (ROS), unstable and reactive molecules formed by one-electron transfers from a redox donor to molecular oxygen. The first of such products is anion superoxide (O_2_^−^), which can be converted to hydrogen peroxide (H_2_O_2_) by superoxide dismutase enzymes. Metal-catalyzed oxidation of H_2_O_2_ can then originate hydroxyl radicals (HO^∙^), the most reactive ROS. Redox balance, that is, regulated production of ROS, is essential for normal cellular physiology, as deregulation in the production of oxidative species, that is, oxidative stress, causes DNA damage, lipid peroxidation, and aberrant posttranslational modification of proteins, thus leading to injury, cell death, and disease [1]. Conversely, accumulating evidence indicates that physiological concentrations of ROS are necessary to support redox signaling events that are involved in important physiological functions and adaptive cell responses, such as chemotaxis, hormone synthesis, immune response, cytoskeletal remodeling, and calcium homeostasis [2]. To be considered as signaling biological messengers, ROS should meet precise spatial and regulatory criteria; that is, they should be produced enzymatically and their levels should be regulated by intracellular molecular mechanisms [3, 4]. Therefore, O_2_^−^ and H_2_O_2_, particularly H_2_O_2_ that is more stable than O_2_^−^ and can cross membranes, are considered signaling molecules because their levels under normal conditions remain under a physiological threshold and their synthesis is enzymatically regulated, whereas HO^∙^ is deemed to be a highly toxic reactant leading to permanent modifications of target molecules that can impact cellular function and life. In the cell, rapid and efficient conversion of H_2_O_2_ to HO^∙^ through the Fenton reaction requires the presence of transition metals, that is, copper and iron. While copper is a more efficient catalyst, iron is much more abundant and thus it is the actual key player in the so-called metal-catalyzed oxidative reactions [5].

While metal-dependent ROS production has historically been associated with necrosis, a form of passive cell death characterized by dissolution of cellular structures, new evidence suggests a role in regulated necrosis. In fact, a form of regulated cell death, termed ferroptosis because it requires iron, has been recently identified [6]. Interestingly, ferroptosis is induced following inhibition of cystine import and downstream glutathione synthesis, leading to the accumulation of intracellular ROS and lipid peroxidation [7]. Further evidence for the role of iron-mediated disruption of cellular redox homeostasis in ferroptosis was provided by the essential regulatory function in ferroptotic cell death of glutathione peroxidase [8], which is the only known enzyme able to reduce phospholipid hydroperoxides and thus one of the most important antioxidant enzymes, and by the demonstration that transferrin-mediated iron import is essential for ferroptosis [9]. Moreover, in hepatocellular carcinoma cells, knockdown of H ferritin, one of the two subunits that compose the iron storage protein ferritin, promoted ferroptosis in response to classical inducers [10].

**Figure 1:**
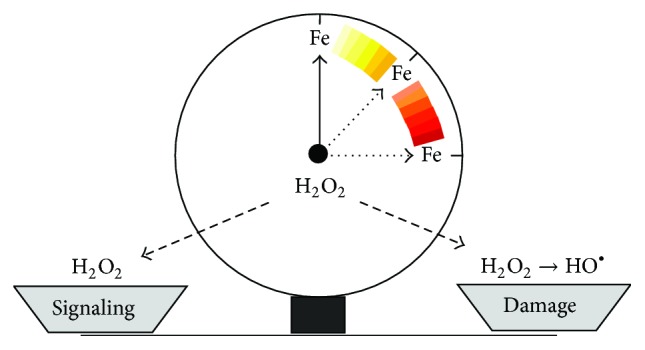
Dual role of ROS as signaling and toxic molecules, iron tips the scale for damage. By triggering Fenton chemistry, increased iron (Fe) availability may change the role of H_2_O_2_ from a relatively safe compound involved in cell signaling to a source of the highly toxic HO^∙^.

During the slow and long process of oxygen accumulation that led to the present 20.9% atmospheric oxygen content initiated about 1.5 billion years ago by photosynthesizing cyanobacteria, all life forms evolved mechanisms to exploit the chemical reactivity of iron for efficient aerobic reactions. Indeed, the number of iron-containing enzymes is striking and includes proteins of vital physiological significance involved in functions such as oxygen transport, cell respiration, and DNA synthesis [11, 12]. Living organisms were also forced to concurrently evolve protection systems against highly damaging HO^∙^ radicals produced by the iron-catalyzed conversion of superoxide and H_2_O_2_. To this aim, a complex regulatory pathway formed by a variety of proteins that bind, transport, and store iron has been developed, in order to maintain an appropriate iron balance in both the individual cells and the whole body [13, 14]. Over the last years, the mechanisms by which iron homeostasis at the cellular and organismal levels is regulated have been elucidated. In this review, we have summarized recent advances in the control of iron homeostasis and how changes in availability of poorly liganded ferrous iron are related to ROS production. Moreover, we propose that whenever the efficiency of the network controlling iron balance is compromised, the role of ROS switches from signaling to damage (Figure 1).

## 2. The IRE/IRP Regulatory Pathway Controls Cellular Iron Homeostasis

**Figure 2:**
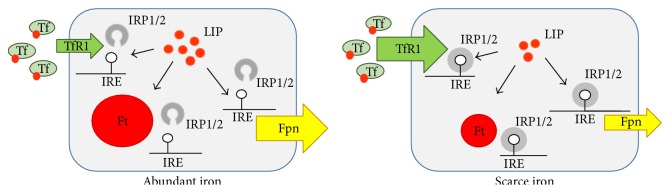
Simplified model of IRP-dependent regulation of intracellular iron homeostasis. The IRE/IRP machinery posttranscriptionally controls the expression of the major proteins of intracellular iron import (transferrin receptor, TfR1), export (ferroportin, Fpn), and storage (ferritin, Ft). Under conditions of iron excess in the labile iron pool (LIP), IRPs lose their RNA-binding capacity, and hence TfR1 mRNA is degraded (small arrow) while Fpn and Ft mRNAs are actively translated (big arrow and circle, resp.). The opposite occurs under conditions of iron deficiency: IRP1 and IRP2 binding to iron responsive elements (IREs) stabilize TfR1 mRNA (big arrow) and prevent the translation of Fpn and Ft mRNAs (small arrow and circle, resp.); this response increases iron availability.

The challenging task of maintaining intracellular iron levels sufficient for essential cellular functions, including ROS-dependent cell signaling, but as low as possible to avoid ROS-mediated injury, is controlled at multiple steps but primarily accomplished by iron regulatory proteins (IRP1 and IRP2), which strictly control intracellular iron metabolism by posttranscriptionally regulating the coordinated expression of proteins involved in iron utilization (e.g., erythroid 5 aminolevulinic acid synthase, mitochondrial aconitase, and* Drosophila* succinate dehydrogenase), uptake (transferrin receptor (TfR1) and divalent metal transporter (DMT1)), storage (H and L ferritin subunits), and export (ferroportin) [15, 16] (Figure 2). IRP1 and IRP2 recognize and bind conserved 25–30 nucleotides-long RNA stem-loop structures named iron responsive elements (IREs) in the untranslated regions of the mRNAs coding for these proteins. It has also been shown that IRPs can bind the mRNAs for other proteins not directly related to iron homeostasis [15, 16].

The activity of IRP1 and IRP2 is dictated by the size of the cellular labile iron pool (LIP), a pool of iron in the low *μ*M range bound to low molecular weight compounds like citrate or glutathione [17], which is in continuous equilibrium with the sites of iron utilization or storage. When cells are iron deficient and thus the size of the LIP is shrunk, IRP1 and IRP2 bind to IREs located in the 3′ region of transcripts and stabilize the mRNA for TfR1 and DMT1, thus increasing the uptake of both transferrin-bound and unbound iron. At the same time, binding to 5′ IRE impairs translation of mRNAs for ferroportin, the only iron exporter, and ferritin, which sequesters iron in a catalytically inactive form. This coordinate regulation eventually expands the cellular LIP. Conversely, when the LIP is large, the IRE-binding activity of both IRPs is decreased, resulting in efficient translation of ferritin and ferroportin mRNAs and lower stability of TfR1 and DMT1 mRNAs, ultimately enhancing iron storage and release over uptake [15, 16] (Figure 2).

Both IRPs are homologous to the mitochondrial TCA cycle enzyme aconitase that converts citrate to isocitrate using an iron sulfur cluster (4Fe–4S) as a cofactor, but only IRP1 can assemble a cluster when sufficient iron is available, thus functioning as cytosolic aconitase, which is the prevailing form in most cells. Conversely, in conditions of iron deficiency, the cluster is disassembled and the IRP1 apoform binds IRE [15, 16]. IRP2 accumulates in iron-deficient cells where it binds IRE motifs with affinity and specificity similar to that of IRP1, whereas in iron-replete cells it is rapidly targeted for proteasomal degradation by an E3 ubiquitin ligase complex that comprises FBXL5, a recently identified protein containing an hemerythrin-like domain that is involved in its regulation according to iron (and oxygen) availability [18]. Under conditions of iron scarcity, the assembly of the di-iron center in the hemerythrin-like domain is impaired and FBXL5 is polyubiquitinated and degraded by the proteasome, thereby leading to IRP2 stabilization. Conversely, when iron is abundant, FBXL5 levels in the cell increase promoting IRP2 (and apo-IRP1) proteasomal degradation [19, 20].

The respective role and importance of IRPs have been revealed by studies involving gene deletion [21, 22]. Ablation of both IRP1 and IRP2, which are ubiquitously expressed, is early embryonic lethal whereas single knockout mice are viable, indicating that the two IRPs can compensate for each other and confirming the in vitro data which suggested essential but largely overlapping functions. However, early studies did not show an apparent phenotype in IRP1 single knockout mice, whereas hematopoietic defects and late onset neurodegeneration were described in mice lacking IRP2. These findings showing a dominant role for IRP2 in the regulation of iron homeostasis in mice were in line with a study indicating that IRP2 is the major regulator of intracellular iron metabolism in humans [23]. However, recent results showed that IRP1 predominantly binds specific IRE-containing mRNAs, such as those coding for erythroid aminolevulinate synthase and hypoxia inducible factor 2*α* (HIF2*α*) [24].

Although iron is the major regulator of the IRE/IRP network, other signals and conditions can modulate the activity of both IRPs, including oxygen tension and nitric oxide (reviewed in [16]). Not surprisingly given the relationship between ROS and iron, IRPs are both targets and modulators of free radical reactions and IRP activity is altered under conditions of oxidative stress. Despite reports showing IRP1 activation in cells exposed to H_2_O_2_, which may be a phenomenon more correlated to the signaling role of H_2_O_2_ rather than to oxidative stress, it appears that IRP1 inactivation occurs in response to ROS both in vitro and in vivo [15, 16]. Overall, since IRP2 is highly susceptible to ROS-mediated downregulation, it is possible to conclude that inhibition of IRP1 and IRP2 binding activity, with ensuing ferritin induction and reduction of LIP, may represent a protective strategy to prevent amplification of oxidative injury (discussed in [25]). The role of ferritin as an antioxidant protein is underscored by the multiplicity of mechanisms leading to its upregulation in response to oxidative challenge; in fact, it has been demonstrated that stressful conditions transcriptionally activate ferritin expression in various cell types, and ferritin overexpression protects from oxidative stress [26].

Readers can refer to recent excellent and comprehensive reviews for specific aspects of the IRE/IRP regulatory network [24, 27–29].

## 3. Conditions in Which Disruption of Cellular Iron Homeostasis Leads to Oxidative Damage 

### 3.1. Neurodegenerative Disorders

Abundant evidence shows that a number of neurodegenerative disorders are characterized by regional iron accumulation in particular areas of the central and/or peripheral nervous systems [30, 31]. This is often caused by cellular iron redistribution and may result in iron-catalyzed Fenton chemistry. For instance, increased iron levels in specific regions of the brain are a hallmark of Parkinson disease [32].

Friedrich's ataxia (FRDA) is a paradigmatic example because the disruption of iron homeostasis in this disease has been well defined [33]. FRDA, which is the most prevalent form of hereditary ataxia in Caucasians, is characterized by progressive degeneration of large sensory neurons in the central and peripheral nervous systems leading to neurological impairment. In addition to neurological symptoms like spinocerebellar and sensory ataxia, FRDA patients also suffer from important nonneurological manifestations, in particular hypertrophic cardiomyopathy. The disease results from loss of function mutations (most often triplet expansion) in the FXN gene that lead to decreased expression of frataxin, a mitochondrial iron-binding protein that interacts with proteins involved in the mitochondrial Fe-S cluster biogenesis [34]. In patients, frataxin deficiency results in disruption of Fe-S cluster biosynthesis, severe mitochondrial iron overload, a hallmark of Fe-S defects, and increased sensitivity to oxidative stress [35].

Although several studies provided evidence that ROS generated through Fenton reaction play a role in FRDA, the primary involvement and the importance of ROS in the pathophysiology of FRDA are still debated [33]. However, the protection afforded by mitochondrial ferritin, which has a strong antioxidant role [36], in yeast, mammalian cells, and fibroblasts from FRDA patients was accompanied by reduced ROS level, thus strongly indicating the involvement of toxic free radicals [37]. Since mitochondrial ferritin plays a protective role in several pathological conditions by sequestering iron [36], these data suggest that iron chelators are of particular interest as therapeutic approaches for FRDA. However, FRDA and other regional sideroses (i.e., iron accumulation in particular tissues or cell compartments) will require novel chelation modalities [38].

### 3.2. Role of Iron and ROS in Anthracycline Cardiotoxicity

The role of iron in anthracycline cardiotoxicity is another illuminating example of the complex interplay and synergism between iron and free radicals as causative factors of apoptosis or other forms of cell damage. Doxorubicin (DOX) is an antineoplastic drug of the anthracyclines family, which plays a recognized key role in the chemotherapy for several types of cancer. However, anthracyclines have established risks of cardiotoxicity, as their chronic administration induces cardiomyopathy and congestive heart failure [39]. This dose-dependent side effect limits the clinical use of DOX in cancer patients. Multiple mechanisms of cardiotoxicity induced by DOX have been described, but activation of the mitochondrial intrinsic pathway of apoptosis seems to represent a major response to anthracycline treatment. The development of anthracycline-induced cardiomyopathy has been found to depend on drug metabolism [40]; in fact, in addition to the role of reductive activation of the quinone moiety of DOX discussed below, a correlation exists between toxicity and myocardial accumulation of anthracycline secondary alcohol metabolites [39]. Conversely, anthracycline oxidative degradation may serve as a salvage pathway for diminishing the levels and toxicity of DOX in cardiomyocytes (reviewed in [41]).

The involvement of iron in DOX-induced cardiac damage is well established, and cardiotoxicity induced by DOX may occur at lower cumulative doses under conditions of iron overload [42]. The adverse role of iron has been suggested by several lines of evidence: in particular, a number of studies showed the protecting efficacy of iron chelators both in patients and in animal models, while others demonstrated that primary and secondary iron overload exacerbated the cardiotoxic effects of the drug, but the underlying molecular mechanisms remain to be fully understood (see [39, 41, 42] for review). Iron has been proposed to act as a catalyst of ROS formation in reactions primed by DOX. In fact, DOX is a redox compound, as NAD(P)H reductases catalyze one-electron reduction of the quinone moiety of the tetracycline ring to the semiquinone free radical, which can regenerate the parent quinone reacting with molecular oxygen. The latter reaction generates O_2_^−^ and its dismutation product H_2_O_2_, which can then be transformed into the more potent HO^∙^ by reactions catalyzed by iron. In turn, HO^∙^ can damage DNA and proteins and initiate membrane lipid peroxidation, thus leading to cardiomyocyte death [42, 43].

However, since antioxidants did not offer protection in clinical settings, the apparently obvious explanation for the aggravating role of iron in DOX cardiotoxicity based on increased iron-catalyzed ROS formation has been called into question [39, 41]. In line with this view, we showed that DOX doses in the range of the plasma levels found in patients undergoing chemotherapy were able to cause apoptotic death of cardiac-derived H9c2 myocytes in the absence of ROS production [44]. Moreover, we provided evidence that activation of the HIF pathway contributes to the cardioprotective effect of the iron chelator dexrazoxane, thus suggesting that the protective capacity of iron chelators against DOX toxicity may be mediated by mechanisms not related to the prevention of ROS formation [45].

We also showed that anthracycline cardiotoxicity is related to ROS-dependent and ROS-independent disruption of cardiac iron homeostasis due to targeted interaction of DOX with IRP1, which leads to a “null” IRP1 devoid of both its functions and hence it is unable to sense iron levels and to regulate iron homeostasis. Moreover, DOX triggers IRP2 degradation, which may serve as a protective role by favoring iron sequestration in newly formed ferritin [25, 46]. Indeed, it has been shown that ferritin is induced in H9c2 cardiomyocytes [47] and mouse hearts [48] exposed to DOX and protects cardiac cells against iron toxicity. Moreover, ferritin H chain plays an important role in the preventive effect of metformin against DOX cardiotoxicity in isolated cardiomyocytes [49]. DOX treatment also results in iron storage by inducing mechanisms leading to higher accumulation of iron into ferritin [50]. Overall, these results suggest that the role of iron in anthracycline-dependent cardiotoxicity may extend beyond the formation of ROS.

On the other hand, recent results regarding mitochondrial ferritin (FtMt), a ferritin type particularly expressed in mitochondria-rich tissues, including the heart, where it prevents iron-mediated oxidative damage [51], reinforce the idea that ROS are involved in the mechanisms linking iron and anthracycline cardiotoxicity. FtMt expression was induced in the heart of mice exposed to DOX [52], and FtMt-deficient mice exposed to DOX are more sensitive to ROS-mediated heart damage and death [53]. In addition, mice with heart-specific deletion or overexpression of ABCB8, which exports iron out of the mitochondria, were more sensitive or resistant, respectively, to DOX cardiotoxicity [52].

The importance of methodological aspects introduces some cautionary issues that should be taken into account when considering the discrepancies reported above regarding the pathophysiological relevance of iron-mediated ROS production in DOX toxicity. In fact, one should keep in mind that the model systems in which the mechanisms of DOX cardiotoxicity have been characterized have inherent limitations in representing the human chronic cardiomyopathy. Moreover, the dual role of ROS in signaling events and cell damage should be considered when evaluating if iron contributes to chronic cardiomyopathy by mechanisms not related to its ability to generate HO^∙^.

## 4. Systemic Iron Metabolism

Given the dual role of iron, elegant control mechanisms have evolved to maintain appropriate body iron levels by means of a complex network of transporters, storage molecules, and regulators. Intestinal iron absorption and iron recycling in reticuloendothelial cells are coordinately orchestrated in order to maintain iron levels in the circulation adequate for the needs of the various tissues and organs but insufficient to activate dangerous ROS production [13, 14].

**Figure 3:**
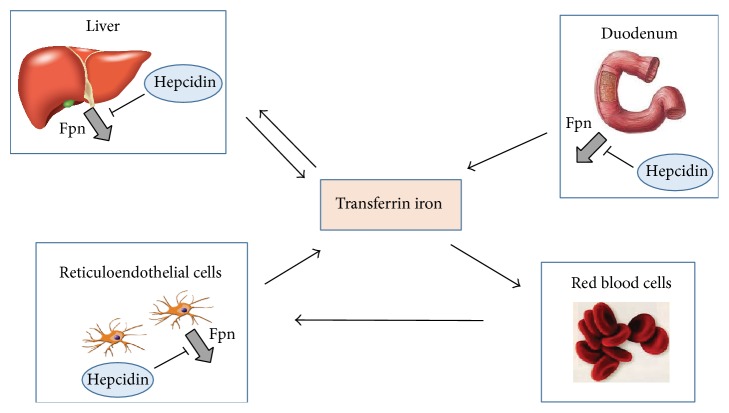
Hepcidin regulates transferrin-mediated body iron traffic. The interaction of hepcidin with ferroportin inhibits the flow of iron into plasma and thereby regulates the transferrin-mediated distribution of iron in the body from sites of iron absorption (duodenum) and recycling (reticuloendothelial cells in spleen and liver) to tissues where it is used (e.g., for the synthesis of hemoglobin in red blood cells) or stored (e.g., liver).

The task of keeping circulating iron in a safe but readily available form is performed by transferrin (Tf), a protein synthesized and secreted by the liver that binds up to two ferric iron atoms with high affinity and is the major iron transport protein. Under physiological conditions, Tf-bound iron is the main source of iron for the majority of tissues, primarily for bone marrow erythroid precursors, which consume iron for hemoglobin biosynthesis (Figure 3).

Tf incorporates iron coming from two major sources: dietary iron (both inorganic and heme iron) absorbed in the duodenum to compensate for daily iron loss and iron derived from destruction of old and effete erythrocytes by reticuloendothelial cells in the spleen and liver (Figure 3). After reduction by the reductase DcytB, dietary iron is transported across the apical membrane of absorptive epithelial cells by DMT1 [54]. In intestinal enterocytes, most iron is then exported to the blood at the basolateral surface by ferroportin, assisted by the function of oxidases (circulating ceruloplasmin and membrane-bound hephaestin) that convert ferrous iron to ferric iron and thus permit the incorporation of iron into Tf. Although various transporters have been identified [54], the mechanisms of intestinal uptake and release of heme iron are less clearly understood, but the majority of heme is degraded in the enterocytes and iron is released by ferroportin, as ferroportin-deficient mice are not viable [55].

Spleen and liver macrophages specialized in recycling iron obtained from the phagocytosis and destruction of senescent erythrocytes are the main iron supplier for hemoglobin synthesis. The major pathway of heme iron recycling involves hemoglobin degradation by cytosolic heme oxygenase-1 and export of heme-derived iron into the circulation by ferroportin [56].

Recent studies in mice with disrupted IRP1 and/or IRP2 in the entire organism or specific tissues have shown that IRPs are important regulators also of systemic homeostasis [22], but hepcidin, a peptide hormone produced and secreted by the liver, can be considered the key regulator of body iron balance [57, 58]. That hepcidin is most important regulator of body iron homeostasis which is also indicated by a number of studies showing that disruption of hepcidin regulation is involved in a variety of disorders associated with iron deficiency (e.g., anemia) or overload (e.g., siderosis). In particular, inadequate hepcidin levels in relation to body iron stores characterize most hereditary iron overload diseases [59]. Hepcidin controls plasma iron concentration and body iron balance by regulating the expression of ferroportin, the only known cellular iron exporter (Figure 3). The binding of hepcidin to ferroportin on the plasma membrane induces its internalization and degradation, thereby blocking iron release [60]. Hepcidin expression is regulated at multiple levels: the expression of hepcidin is induced by iron overload, inflammatory stimuli, or endoplasmic reticulum stress [57, 58, 61]; this mechanism stops the efflux of unwanted iron in the circulation by negatively modulating iron absorption by enterocytes, iron recycling by reticuloendothelial cells, and iron mobilization from hepatic stores. Conversely, increased erythropoietic activity under conditions of iron deficiency, anemia, and hypoxia represses hepcidin, thereby leading to higher iron availability for new erythrocytes synthesis [20, 62]. Among the positive regulators of hepcidin, iron exerts its effect through the BMPs/SMAD dependent pathway, while inflammatory cytokines, especially IL-6, activate the JAK2-STAT3 signaling cascade. Erythroferrone, produced by erythroid precursors in the marrow and the spleen in response to erythropoietin, seems to be the major inhibitor of hepcidin production when erythropoiesis is stimulated [63].

### 4.1. Iron Overload Conditions

**Figure 4:**
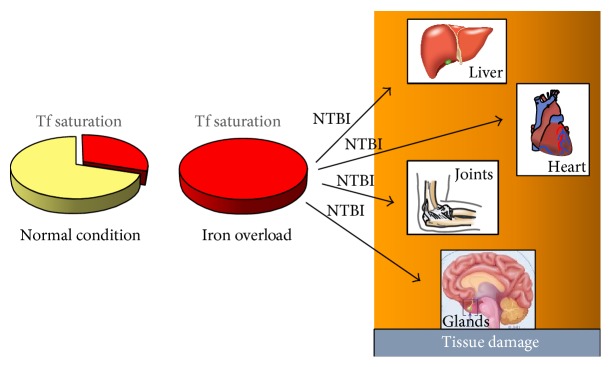
Mechanisms of iron overload mediated tissue damage. Whenever the iron-binding capacity of transferrin (Tf) is exceeded, non-transferrin-bound iron (NTBI) forms, penetrates into the cells, and undergoes redox cycling, ultimately leading to cell injury and organ damage.

Two mechanisms protect the cells from the damaging effects of excess body iron: the partial saturation of Tf at the systemic level and the IRP-dependent regulated expression of TfR1 at the cellular level. Under normal conditions, Tf saturation is around 30% and the protein can therefore bind excess iron entering the circulation, thus functioning as a protective shield against HO^∙^ production. However, under conditions of heavy systemic iron overload (primarily due to insufficient hepcidin production), the iron-binding capacity of Tf is exceeded, and non-transferrin-bound iron (NTBI) is formed (Figure 4). Despite the downregulation of TfR1, cells become iron-loaded because NTBI, that is, iron associated with various low molecular weight plasma components, like citrate, phosphates, and proteins, lacks a regulated uptake system and hence is able to penetrate into the cells [38]. The consequent decrease in IRP binding activity leads to efficient ferritin mRNA translation to promote iron storage, but ultimately the high capacity of ferritin is exceeded as this unabated flux of NTBI continues. NTBI is clearly involved in tissue siderosis, but the incomplete characterization of the iron species involved prevents a clear understanding of how it enters the cell under different pathological conditions. NTBI enters into cells mainly through divalent cation transporters such as DMT1 and ZIP14, which require previous reduction to ferrous iron [64, 65] or Ca^2+^ channels, either L-type or T-type, particularly in cardiomyocytes [66]. NTBI toxicity within cells derives from the fact that iron in the expanded LIP is no longer protected from redox cycling; hence, it accelerates the catalysis of reactions that produce HO^∙^, which then cause lipid peroxidation and organelle damage and ultimately cell death (Figure 4). In particular, mitochondria appear to be primarily affected by iron-mediated HO^∙^ production. In fact, it has been demonstrated that mitochondria rapidly acquire NTBI, and this, along with their high generation of ROS [67], props up oxidative damage. Apparently, the variety of enzymatic and nonenzymatic antioxidant defense systems is inadequate to prevent metal-catalyzed oxidative injury once iron in the LIP is increased [66]. Although production of hydroxyl radical and lipid peroxidation are important in the initiation of iron overload pathology, additional mechanisms involving apoptosis and fibrosis can account for its complex pathophysiology that leads to organ failure [58].

The most important clinical conditions involving primary and secondary iron overload leading to iron-mediated tissue damage are genetic hemochromatosis and transfusional siderosis, respectively. Hereditary hemochromatosis linked to mutations in HFE, a MHC class I-like protein that is a necessary component of the iron-sensing machinery controlling hepcidin expression, is the most common genetic disease in Caucasians and presents a multisystem involvement: although iron overload first affects the liver, in hemochromatosis patients endocrine abnormalities, cardiac problems, and arthropathy are also common [68]. Loss of HFE function leads to inappropriately low hepcidin production and unneeded iron release in the bloodstream from the duodenum and reticuloendothelial system. Consequent NTBI formation and iron deposition in parenchymal cells lead to oxidative damage and determine the clinical features of hemochromatosis [69].

Secondary iron overload is mainly observed in association with transfusion-dependent diseases [70]. Since our body lacks any regulated mechanism to effectively excrete excess iron, long-term blood transfusion inescapably results in iron overload in patients. Transfusional iron overload affects particularly patients with inherited hemoglobinopathies, such as *β* thalassemia, which is the secondary iron overload condition more closely linked to tissue iron overload [71, 72]. However, the adverse effects of iron overload are also found in patients with a variety of conditions (e.g., Blackfan-Diamond anemia, aplastic anemia, sideroblastic anemia, myelodysplasia, etc.).

With continued transfusion, reticuloendothelial cells can no longer safely store all the excess iron, which thus enters the circulation in amounts that exceed the binding capacity of Tf, and NTBI develops [69]. It should be noted that in conditions such as *β* thalassemia hepcidin levels are paradoxically low because erythropoiesis-dependent downregulation prevails over the upregulation associated with body iron levels; therefore, high intestinal iron absorption contributes to iron overload [20, 62]. While in hemochromatosis patients excess iron is removed by phlebotomy, the treatment of transfusional iron overload is mainly based on iron chelation therapy [61]. Iron chelators can be considered as antioxidants, but not all the possible molecules able to bind iron can be regarded as safe antioxidants. Since all the six iron atoms have to be bound to the chelator to form a stable complex, incomplete iron-chelate complexes (e.g., iron-EDTA) can undergo redox cycling and generate harmful free radicals. Therefore, for efficient and safe scavenging of excess iron, chelating molecules and/or dosages should be carefully evaluated to raise the thermodynamic stability of the iron-ligand complex.

## 5. Conclusions

Presently, it is not completely clear as to why in some conditions ROS are associated with cell damage and scavenging high ROS levels improves metabolic homeostasis, whereas in other settings ROS exert signaling functions important for essential cell activities. Possible explanations for these distinct biological specificities of ROS action include differences in the amount, sources, duration, and localization of ROS production; in addition, certainly, the higher ROS reactivity is, the greater toxicity is, while signaling capacity is diminished. Therefore, H_2_O_2_, which is relatively stable and diffusible, is indeed a mild oxidant but is suitable for signaling. However, in the presence of ferrous iron, H_2_O_2_ can generate the highly reactive and toxic HO^∙^. Therefore, we propose that increased availability of iron not bound to proteins specifically evolved to transport or store this essential metal can make the critical difference between the two opposite functions of ROS. The view that iron plays a main role in the scenario leading ROS to become signal or stress agents is supported by the sophisticated systems regulating intracellular and systemic iron homeostasis that we have summarized in this review. Additional evidence indicating the contribution of iron emerges from the number of studies showing the occurrence of ROS-mediated tissue damage whenever the control of iron metabolism is disrupted, conditions of which we selectively highlighted a few illustrative examples. Improved understanding of the complex interplay between iron metabolism and redox homeostasis will clarify these pathways and their relevance in pathophysiology. In consideration of the disappointing results of antioxidant therapy for a variety of diseases [73], targeting iron will possibly represent a more advanced therapeutic approach aimed at preventing the harmful effects of ROS while permitting their physiological role in cell signaling.
